# Prognostic Value of the Number of Removed Lymph Nodes in Vulvar Squamous Cell Carcinoma Patients With Node-Positive Disease: A Population-Based Study

**DOI:** 10.3389/fonc.2018.00184

**Published:** 2018-05-30

**Authors:** San-Gang Wu, Wen-Wen Zhang, Jia-Yuan Sun, Qiong-Hua Chen, Zhen-Yu He, Juan Zhou

**Affiliations:** ^1^Department of Radiation Oncology, Xiamen Cancer Hospital, The First Affiliated Hospital of Xiamen University, Xiamen, China; ^2^Department of Radiation Oncology, Sun Yat-sen University Cancer Center, State Key Laboratory of Oncology in South China, Collaborative Innovation Center of Cancer Medicine, Guangzhou, China; ^3^Department of Obstetrics and Gynecology, The First Affiliated Hospital of Xiamen University, Xiamen, China

**Keywords:** vulvar cancer, lymphadenectomy, removed node count, survival, surveillance, epidemiology, and end result

## Abstract

**Introduction:**

To investigate the effect of the number of removed lymph nodes (RLNs) on outcomes in patients with node-positive vulvar squamous cell carcinoma (SCC).

**Methods:**

This population-based retrospective study included vulvar SCC patients recorded on the surveillance, epidemiology, and end results database, who received surgery and lymphadenectomy. Cox regression proportional hazards were used for multivariate analysis. The number of RLNs was examined as a 4-level categorical variable based on quartiles.

**Results:**

In total, 703 patients were identified. Patients with a higher RLN count had a significantly higher number of positive lymph nodes. The 3-year cause-specific survival (CSS) rates were 48.9, 65.9, 73.1, and 67.3% in patients with 1–6, 7–10, 11–16, and 17–45 RLNs, respectively (*p* < 0.001), and the 3-year overall survival (OS) rates were 36.1, 50.6, 61.1, and 57.6%, for the same RLN groups, respectively (*p* < 0.001). RLN count was an independent predictor of outcome. Using 7–10 RLNs as reference, patients with 1–6 RLNs had poor CSS [hazard ratio (HR) 1.727, 95% confidence interval (CI) 1.201–2.485, *p* = 0.003] and OS (HR 1.436, 95% CI 1.078–1.911, *p* = 0.013), while there were comparable outcomes in patients with 11–16 and 17–45 RLNs to patients with 7–10 RLNs. Adjuvant radiotherapy improved CSS (*p* = 0.023) and OS (*p* = 0.003) in patients with ≤6 RLNs, but was not associated with better outcomes in patients with >6 RLNs.

**Conclusion:**

The removal of more than six lymph nodes improves vulvar SCC outcomes in patients with node-positive disease.

## Introduction

Vulvar squamous cell carcinoma (SCC) is a rare gynecological malignancy, accounting for approximately 3–5% of all gynecological cancers (1, 2). Lymph node status is an important indicator for predicting the outcome of vulvar SCC. One meta-analysis showed the 5-year overall survival (OS) rate to be 84.5, 58.5, 47.4, and 30.1% in patients with node-negative disease, one positive lymph node (PLN), two PLNs, and more than three PLNs, respectively (3). The standard treatment procedure for patients with node-positive vulvar SCC is radical inguinal–femoral lymphadenectomy. However, controversy exists regarding the optimal extent of inguinal–femoral lymphadenectomy in patients with vulvar cancer.

Theoretically, removing more lymph nodes reduces the potential for lymph node micrometastases, reducing the risk of relapse and improving survival. Several previous studies have found that in patients with node-negative vulvar SCC, an increased number of removed lymph nodes (RLNs) was significantly associated with better outcomes (4–8). In patients with early-stage vulvar SCC, sentinel lymph node biopsy (SLNB) has been confirmed as safe and feasible (9–11), with no significant difference in clinical outcomes between inguinal-femoral lymphadenectomy and SLNB (12). Therefore, inguinal–femoral lymphadenectomy may be avoided in patients with early-stage vulvar SCC after careful lymph node assessment. A study of a population-based cohort from the surveillance, epidemiology, and end results (SEER) program has confirmed that a higher RLN count found during lymphadenectomy was associated with significantly better disease-specific survival in patients with stage III node-negative vulvar SCC, but not in patients with early-stage vulvar cancer (6).

The number of PLNs is significantly related to survival outcomes in patients with vulvar SCC; therefore, the extent of lymphadenectomy could be a more accurate assessment of lymph node status of patients. However, the optimal extent of lymphadenectomy in patients with node-positive disease remains controversial (13, 14). A population-based study to investigate the role of the RLN count in vulvar SCC is important, due to the rarity of the disease. The purpose of this retrospective population-based study was to assess the effect of RLN count on outcome in patients with node-positive vulvar SCC treated with lymphadenectomy.

## Materials and Methods

### Patients

This population-based study identified vulvar cancer patients diagnosed between 2004 and 2013, using the SEER program. This program, maintained by the National Cancer Institute, covers approximately 28% of the United States population and includes information on the demographics, incidence, and outcomes of specific cancers (15). We have obtained the permission to access the publicly SEER database with the reference number 11025-Nov 2016. We included patients who met the following criteria: (1) histologically confirmed node-positive vulvar SCC who received surgery including lymphadenectomy; (2) the exact number of RLNs and PLNs were recorded; and (3) patient characteristics including age, race/ethnicity, tumor grade, tumor size, and receipt of radiotherapy or chemotherapy were available. Patients who received preoperative radiotherapy or SLNB were excluded. The institutional review board of the First Affiliated Hospital of Xiamen University had approved this study.

The following demographic, clinicopathological, and treatment characteristics were included: age, race/ethnicity, tumor grade, tumor size, receipt of radiotherapy or chemotherapy, and the number of PLNs and RLNs. The primary endpoints were cause-specific survival (CSS) and OS. CSS was defined as time from initial diagnosis to the date of vulvar cancer-related death. OS was defined as time from initial diagnosis to the date of death or last follow-up.

### Statistical Analysis

The χ^2^ test, Fisher’s exact test, and one-way analysis of variance were used to compare the differences in patient characteristics among the RLN groups. Recognizing that the number of RLNs may have been incompletely counted or that there may be natural interindividual variation in lymph node distribution, the variable was examined as a 4-level categorical variable based on quartiles. Survival curves were plotted using the Kaplan–Meier method and compared using the log-rank test. Cox regression analysis was used to identify significant prognostic factors. Variables with *p* < 0.05 in the univariate analysis were entered into multivariate Cox regression models. All statistical tests were conducted using SPSS version 22 statistical software (IBM Corporation, Armonk, NY, USA). A *p* value less than 0.05 was considered statistically significant.

## Results

### Patients Characteristics and Number of RLNs

A total of 703 patients were included and the patient characteristics are displayed in Table 1. The median age of the patients was 69 years (range 21–95 years). Of the patients, 79.1% (*n* = 556) were non-Hispanic White. The median tumor size was 35 mm (range 2–200 mm). A total of 490 (69.7%) and 204 (29.0%) patients were received adjuvant radiotherapy and chemotherapy, respectively.

**Table 1:** The baseline characteristics of 703 vulvar cancer patients.

Variable		Number of RLNs				
	All	1–6	7–10	11–16	17–45	*p*-Value
Age (years) (mean ± SD)	67.0 ± 14.3	69.7 ± 14.7	67.4 ± 15.2	64.8 ± 13.8	66.1 ± 13.2	0.008

**Race**						
Non-Hispanic White	556	139 (76.8)	137 (80.1)	156 (80.4)	124 (79.0)	0.966
Non-Hispanic Black	49	15 (8.3)	10 (5.8)	14 (7.2)	10 (6.4)	
Hispanic (all Races)	76	21 (11.6)	20 (11.7)	19 (9.8)	16 (10.2)	
Other	22	6 (3.3)	4 (2.3)	5 (2.6)	7 (4.5)	

**Tumor grade**						
Well differentiated	98	30 (16.6)	24 (14.0)	21 (10.8)	23 (14.6)	0.534
Moderately differentiated	369	96 (53.0)	82 (48.0)	109 (56.2)	82 (52.2)	
Poorly/undifferentiated	236	55 (30.4)	65 (38.0)	64 (33.0)	52 (33.1)	
Tumor size (mm) (mean ± SD)	39.4 ± 23.2	40.1 ± 20.6	39.6 ± 27.1	38.0 ± 23.2	40.0 ± 21.6	0.819

**Number of PLNs (***n***)**						
Mean ± SD	2.3 ± 2.1	1.7 ± 0.9	2.4 ± 2.0	2.5 ± 2.0	2.9 ± 2.8	<0.001
1	320	98 (54.1)	85 (49.7)	78 (40.2)	59 (37.6)	<0.001
2	174	53 (29.3)	33 (19.3)	52 (26.8)	36 (22.9)	
≥3	209	30 (16.6)	53 (31.0)	64 (33.0)	62 (39.5)	

**Adjuvant radiotherapy**						
No	213	62 (34.3)	55 (32.2)	48 (24.7)	48 (30.6)	0.215
Yes	490	119 (65.7)	116 (67.8)	146 (75.3)	109 (69.4)	

**Chemotherapy**						
No/unknown	499	127 (70.2)	117 (68.4)	144 (74.2)	111 (70.7)	0.659
Yes	204	54 (29.8)	54 (61.6)	50 (25.8)	46 (29.3)	

*PLN, positive lymph node; RLN, removed lymph node*.

**Figure 1:**
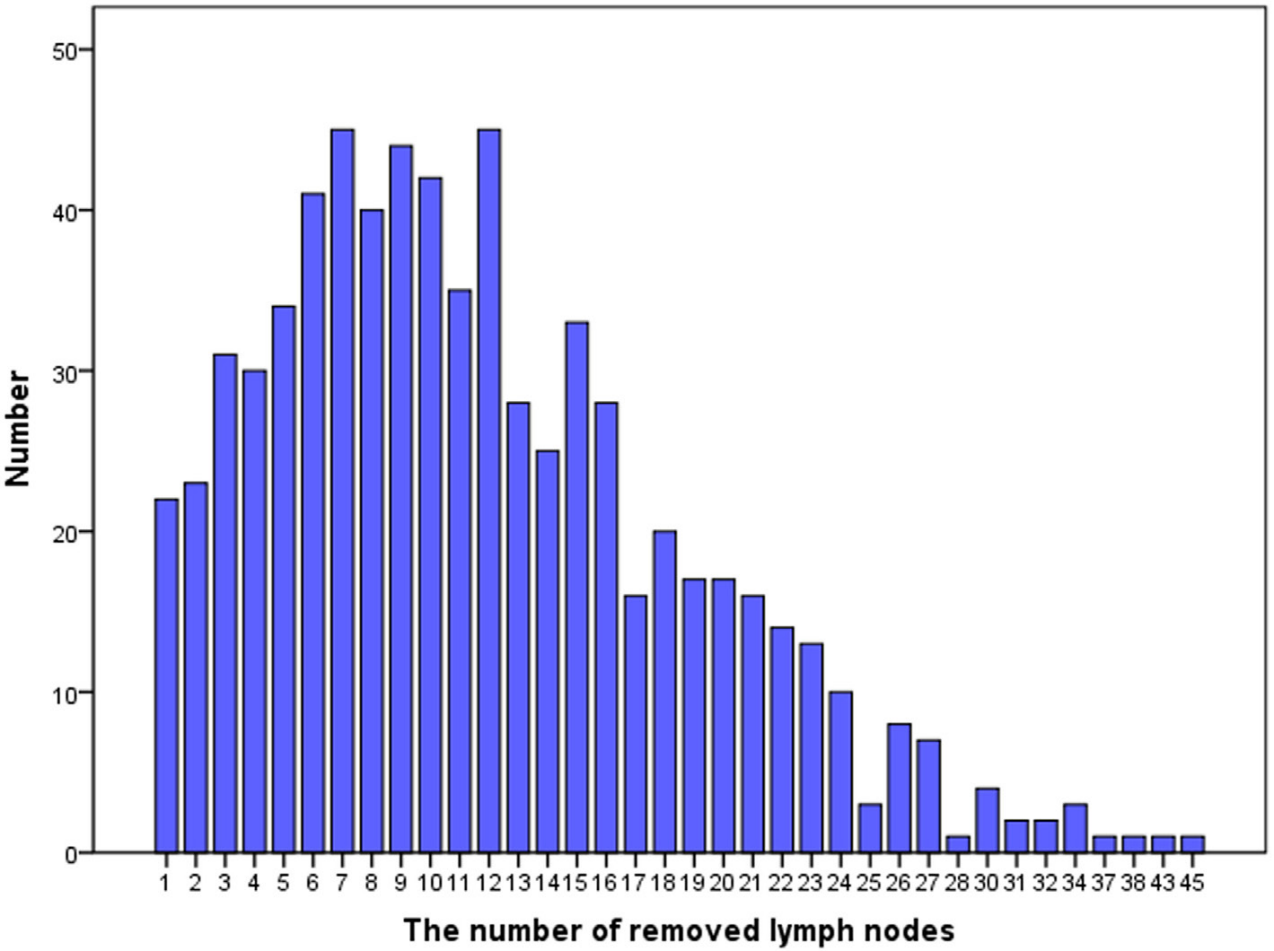
Distribution of number of resected lymph nodes.

The median RLN count was 12 (25th percentile: 6, 75th percentile: 16; range 1–45) and the median PLN count was 2 (range 1–17). Figure 1 shows the distribution of number of RLNs. The number of RLNs was classified using quartiles as follows: Group 1 (1–6, *n* = 181), Group 2 (7–10, *n* = 171), Group 3 (11–16, *n* = 194), and Group 4 (17–45, *n* = 157). Patients with a higher RLN count were more likely to be elderly (*p* = 0.008). In addition, a higher PLN count was seen in patients with a higher RLN count, and Group 2, 3, and 4 patients had a significantly higher number of RLNs compared with Group 1. There were no associations between race/ethnicity, grade, tumor size, and receipt of adjuvant therapy among the PLN groups.

### Correlating the Number of PLNs With Survival

The median follow-up was 21.0 months (range 0–119 months). A total of 344 patients died and 230 (66.9%) of these died from vulvar cancer-related diseases. The 3-year CSS and OS rates were 64.1 and 51.5%, respectively.

**Figure 2:**
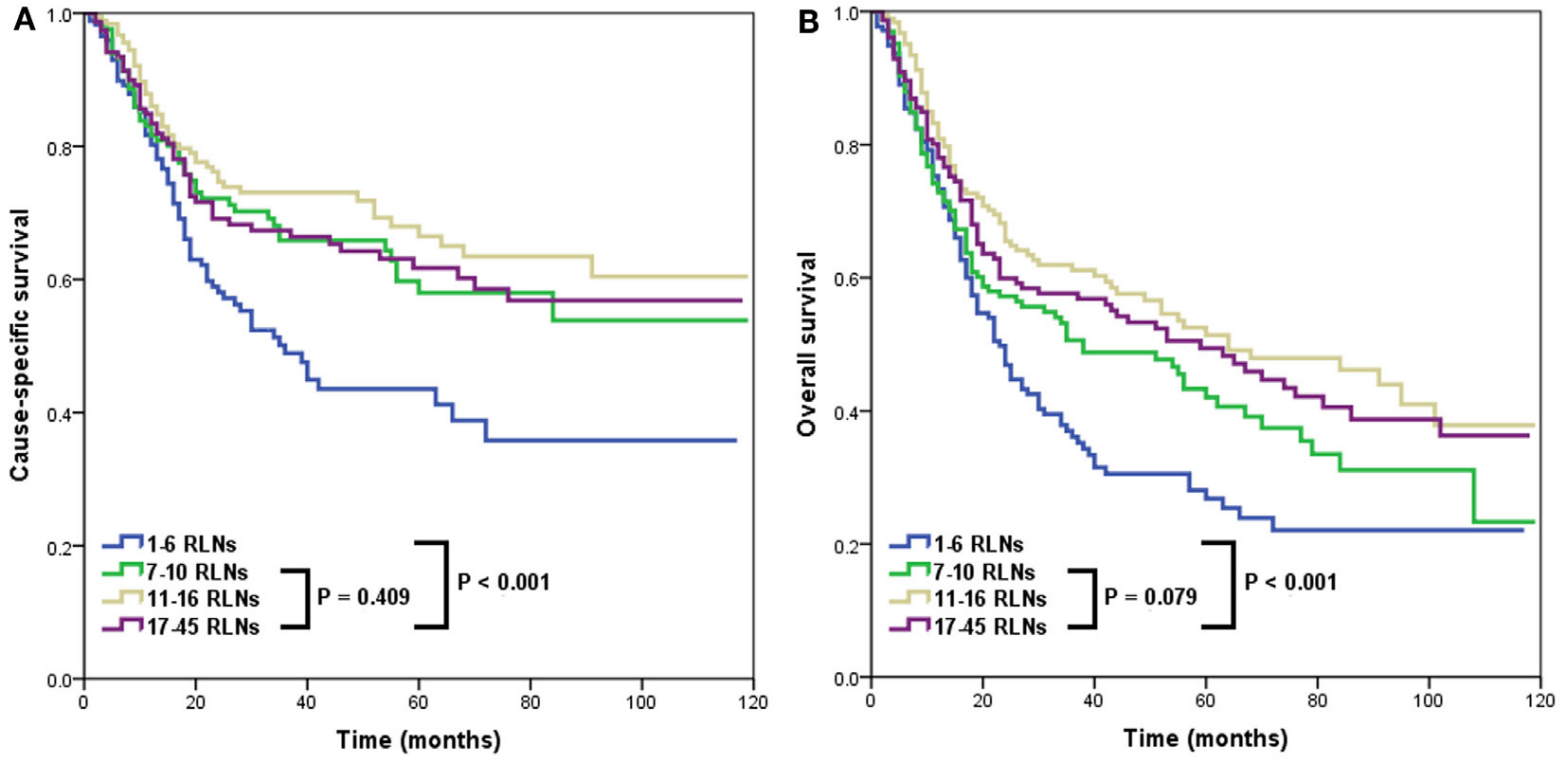
Impact of the number of removed lymph nodes (RLNs) on cause-specific survival **(A)** and overall survival **(B)**.

A higher RLN count was associated with better CSS and OS. In the four categories of RLNs, the 3-year CSS rates were 48.9, 65.9, 73.1, and 67.3% for patients in Groups 1, 2, 3, and 4, respectively (*p* < 0.001) (Figure 2A). The 3-year OS rates were 36.1, 50.6, 61.1, and 57.6% for patients in Groups 1, 2, 3, and 4, respectively (*p* < 0.001) (Figure 2B). However, there was no significant difference in CSS (*p* = 0.409) and OS (*p* = 0.079) among Groups 2, 3, and 4.

### Analysis of Prognostic Factors on Outcomes

The results of Cox regression univariate analyses indicated that age, race/ethnicity, tumor size (continuous variable), PLN count, and RLN count were prognostic factors in CSS and OS. In addition, receipt of radiotherapy or chemotherapy was associated with better CSS and OS (Table 2).

**Table 2:** Univariate Cox regression analysis of prognostic factors of 703 vulvar cancer patients.

Variables	CSS			OS		
	HR	95%CI	*p*-Value	HR	95%CI	*p*-Value
Age (continuous variable)	1.037	1.027–1.048	<0.001	1.041	1.032–1.049	<0.001

**Race**						
Non-Hispanic White	1			1		
Non-Hispanic Black	0.478	0.245–0.932	0.030	0.698	0.444–1.098	0.119
Hispanic (all Races)	0.881	0.581–1.336	0.550	0.898	0.643–1.255	0.529
Other	0.312	0.100–0.977	0.046	0.403	0.179–0.904	0.027

**Tumor grade**						
Well differentiated	1			1		
Moderately differentiated	0.937	0.646–1.358	0.730	0.972	0.719–1.314	0.853
Poorly/undifferentiated	0.852	0.570–1.272	0.433	0.879	0.635–1.217	0.436
Tumor size (continuous variable)	1.013	1.009–1.018	<0.001	1.012	1.008–1.016	<0.001

**Number of PLNs (***n***)**						
1	1			1		
2	1.408	1.007–1.969	0.046	1.156	0.879–1.521	0.3
≥3	2.367	1.753–3.197	< 0.001	2.208	1.744–2.796	<0.001

**Number of RLNs (***n***)**						
7–10	1			1		
1–6	1.588	1.113–2.266	0.011	1.335	1.008–1.767	0.044
11–16	0.782	0.530–1.153	0.215	0.712	0.527–0.961	0.027
17–45	0.962	0.653–1.416	0.844	0.817	0.603–1.107	0.192

**Adjuvant radiotherapy**						
No	1			1		
Yes	0.806	0.612–1.062	0.126	0.721	0.580–0.895	0.003

**Chemotherapy**						
No/unknown	1			1		
Yes	0.917	0.688–1.222	0.556	0.744	0.584–0.947	0.016

*CI, confidence interval; CSS, cause-specific survival; HR, hazard ratio; OS, overall survival; PLN, positive lymph node; RLN, removed lymph node*.

The results of multivariate Cox regression analyses showed that RLN count was an independent predictor for outcomes, with a higher RLN count associated with better CSS and OS. Using an RLN count of 7–10 as reference, patients with 1–6 RLNs had poor CSS [hazard ratio (HR): 1.727, 95% confidence interval (CI): 1.201–2.485, *p* = 0.003] and OS (HR: 1.436, 95% CI: 1.078–1.911, *p* = 0.013), while there were comparable outcomes in patients with 11–16 RLNs and 17–45 RLNs compared with patients with 7–10 RLNs. Age, race/ethnicity, tumor size, and the number of PLNs were also the independent predictors for survival outcomes (Table 3).

**Table 3:** Multivariate Cox regression analysis of prognostic factors of 703 vulvar cancer patients.

Variables	CSS			OS		
	HR	95%CI	*p*-Value	HR	95%CI	*p*-Value
Age (continuous variable)	1.033	1.023–1.044	<0.001	1.039	1.031–1.048	<0.001

**Race**						
Non-Hispanic White	1			1		
Non-Hispanic Black	0.625	0.317–1.233	0.175	0.972	0.610–1.547	0.904
Hispanic (all Races)	0.888	0.581–1.358	0.583	0.930	0.663–1.306	0.677
Other	0.312	0.099–0.980	0.046	0.410	0.182–0.925	0.032

Tumor size (continuous variable)	1.012	1.007–1.017	<0.001	1.011	1.007–1.015	<0.001

**Number of PLNs (***n***)**						
1	1			1		
2	1.280	0.912–1.797	0.153	1.045	0.792–1.379	0.756
≥3	2.390	1.751–3.264	<0.001	2.166	1.696–2.764	<0.001

**Number of RLNs (***n***)**						
7–10	1			1		
1–6	1.727	1.201–2.485	0.003	1.436	1.078–1.911	0.013
11–16	0.864	0.583–1.281	0.468	0.809	0.597–1.096	0.171
17–45	1.023	0.692–1.514	0.908	0.871	0.641–1.183	0.376

**Adjuvant radiotherapy**						
No	–			1		
Yes	–	–	–	0.982	0.774–1.247	0.884

**Chemotherapy**						
No/unknown	–			1		
Yes	–	–	–	0.889	0.675–1.171	0.403

*CI, confidence interval; CSS, cause-specific survival; HR, hazard ratio; OS, overall survival; PLN, positive lymph node; RLN, removed lymph node*.

### Effect of Adjuvant Radiotherapy on Outcomes According to Number of RLNs

**Figure 3:**
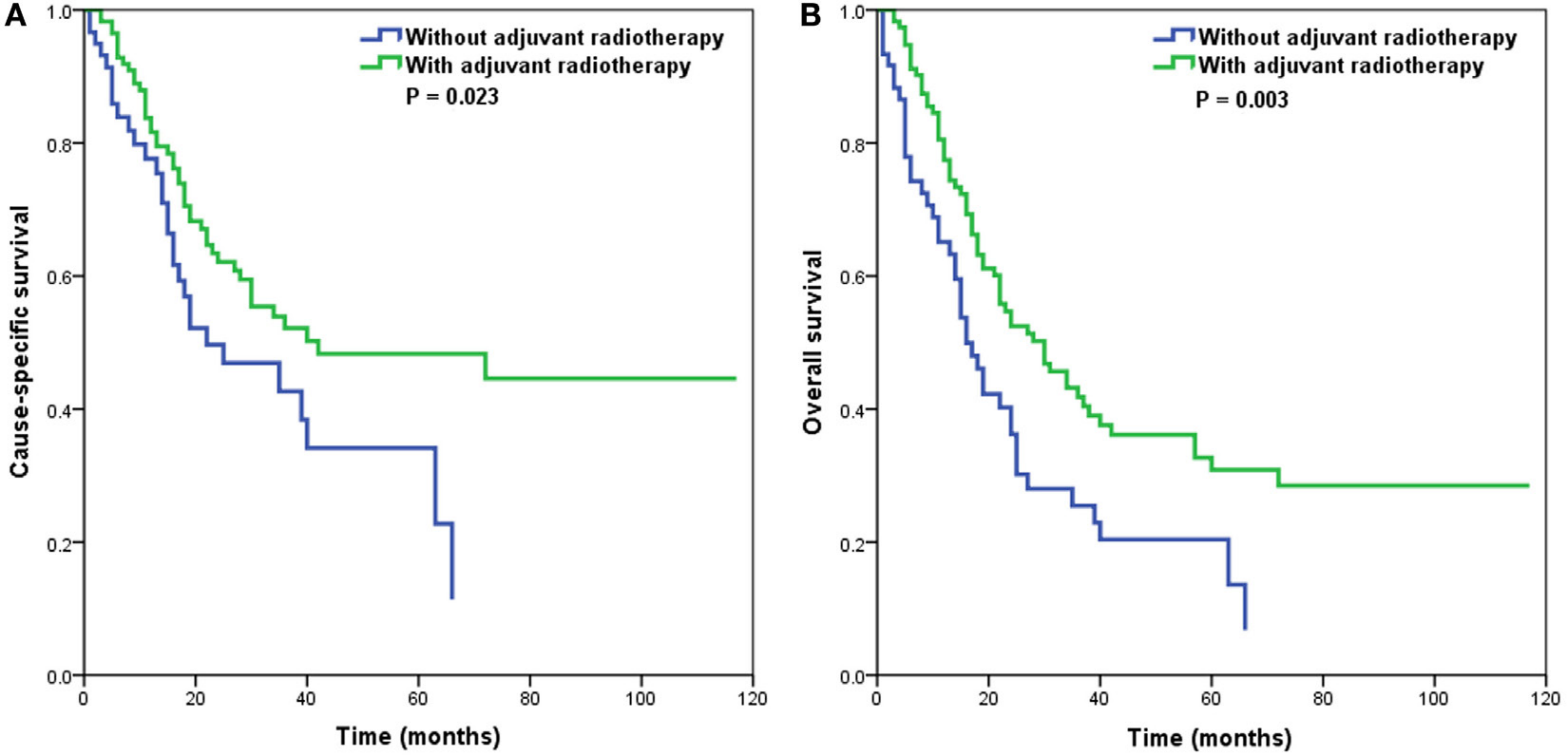
Impact of adjuvant radiotherapy on cause-specific survival **(A)** and overall survival **(B)** in patients with 1–6 removed lymph nodes.

Adjuvant radiotherapy was not an independent predictor in our multivariate analyses. However, several studies have indicated that adjuvant radiotherapy is associated with an improvement of outcomes in patients with node-positive vulvar SCC (16–19). Therefore, we further analyzed the effect of adjuvant radiotherapy on survival outcomes according to the number of RLNs. Our results showed that in patients with ≤6 RLNs, adjuvant radiotherapy was associated with better CSS (*p* = 0.023) (Figure 3A) and OS (*p* = 0.003) (Figure 3B), while adjuvant radiotherapy was not associated with improved CSS (*p* = 0.740) or OS (*p* = 0.097) in patients with >6 RLNs.

## Discussion

In this retrospective study, we used a population-based cohort to investigate clinical outcomes according to number of RLNs in patients with node-positive vulvar SCC, and our results indicated that patients with ≤6 RLNs had a higher risk of death compared with patients with >6 RLNs.

Lymph node status has been confirmed as an important indicator in prognostic assessment, and has been included in the staging system for vulvar SCC (20). A higher number of RLNs may increase the potential for lymph node micrometastases. However, in a study that included 158 vulvar SCC patients who underwent bilateral inguinofemoral lymphadenectomy, Baiocchi et al. found that a higher RLN count did not correlate with lymph node involvement (14). In our study, we observed a significant difference in the PLN count according to various RLN count groups, with a significantly higher number of PLNs found in patients with a higher number of RLNs. Based on our results, we confirmed our hypothesis that a higher RLN count may more accurately predict nodal status in vulvar SCC patients with node-positive disease.

Our results also found that older age was associated with fewer RLNs. The reason for this difference remains unclear. Since all of patients identified in the study underwent surgery and lymphadenectomy, we can presume that these patients were not subject to serious complications and were suitable for surgical treatment and lymphadenectomy. A study by Panici et al. showed that the survival benefit of lymphadenectomy in older patients was similar to that of younger patients, and that lymphadenectomy did not increase the probability of complications (21). Although we could not obtain surgical complication data from the SEER database, several previous studies have confirmed that lymphadenectomy does not have higher complication rates such as wound breakdown and lymphedema in older patients than in younger patients (21–24). These data suggest that complete lymphadenectomy is an important intervention in older patients too.

The optimal number of lymph nodes that should be removed during inguinal lymphadenectomy remains unclear. Baiocchi et al. found that in the 50.6% of study patients with node-positive disease, there was no significant difference in outcomes between patients with <12 RLNs and those with ≥12, while resection of <12 lymph nodes in node-positive disease negatively affected survival outcomes (*p* < 0.05) (14). However, a study by Gill et al. did not find any difference in survival according to number of PLNs in univariate analysis. In our population-based study, we observed that resection of ≤6 lymph nodes had a significantly negative impact on CSS and OS (13).

Adjuvant radiotherapy has been confirmed to improve outcomes in patients with node-positive vulvar SCC in previous research (16–19). A total of 69.7% of the patients in our study underwent adjuvant radiotherapy; however, adjuvant radiotherapy had no effect on survival outcomes of patients, according to the results of multivariate analyses. Very few studies have assessed the value of adjuvant radiotherapy according to the number of RLNs. Parthasarathy et al. studied 208 patients with a single PLN, and their results suggested that adjuvant radiotherapy may improve the disease-specific survival of single PLN patients with ≤12 RLNs (18). A study by Polterauer et al. found that adjuvant radiotherapy only improved survival in patients with a lymph node ratio >20%, and not in patients with a lymph node ratio ≤20% (25). In our series, adjuvant radiotherapy has been statistically shown to improve outcomes in patients who received a less extensive lymphadenectomy (≤6 RLNs), while not affecting the survival outcomes of patients with ≥7 RLNs. Therefore, assessment of the number of RLNs may not only indicate the potential therapeutic effect of lymphadenectomy, but may also be a useful method of selecting appropriate candidates for adjuvant radiotherapy.

Several limitations of our study should be acknowledged. First, the SEER database lacks a centralized pathology review, causing potential variability and subjectivity in determining the number of RLNs, and patterns of disease recurrence were also not recorded. Second, tumor location, such as midline, whether bilateral groin lymphadenectomy was performed, and the RLN count for each side or per patient was also not available in the SEER program. Third, details of the lymphadenectomy techniques used—debulking of bulky lymph nodes, superficial groin dissection, or superficial plus deep groin dissection—were lacking. Moreover, the cutoff point of the number of RLNs was classified based on quartiles. The optimal cutoff point of the number of RLNs should be explored in future prospective multicenter studies. Many of these limitations could be overcome by evaluating patients attending a single institution, but the rarity of the disease means that the use of a population-based database such as the SEER program, with its inherent strengths and weaknesses, is required.

## Conclusion

In conclusion, our results suggest that RLN count is an independent predictor of outcome in vulvar SCC with node-positive disease. Patients with >6 RLNs had a significantly higher CSS and OS than patients with ≤6 RLNs. Further prospective studies with more patients are needed to further evaluate the role of removal of lymph nodes in vulvar SCC.

## Ethics Statement

The institutional review board of the First Affiliated Hospital of Xiamen University had approved this study.

## Author Contributions

S-GW, W-WZ, Z-YH, and JZ are lead authors who participated in data collection, manuscript drafting, table/figure creation, and manuscript revision. W-WZ, Q-HC, and J-YS aided in data collection. S-GW is the dosimetrist who contributed dosimetric data and figures. S-GW and JZ are senior authors who aided in drafing the manuscript and manuscript revision. Z-YH and JZ are corresponding authors who initially developed the concept and drafted and revised the manuscript. All authors read and approved the final manuscript.

## Conflict of Interest Statement

The authors declare that the research was conducted in the absence of any commercial or financial relationships that could be construed as a potential conflict of interest. The reviewer MS and handling Editor declared their shared affiliation.
